# Remodeling of Retinal Arterioles and Carotid Arteries in Heart Failure Development—A Preliminary Study

**DOI:** 10.3390/jcm11133721

**Published:** 2022-06-27

**Authors:** Janusz Sadowski, Ryszard Targonski, Piotr Cyganski, Paulina Nowek, Magdalena Starek-Stelmaszczyk, Katarzyna Zajac, Judyta Juranek, Joanna Wojtkiewicz, Andrzej Rynkiewicz

**Affiliations:** 1 Department of Cardiology and Internal Medicine, School of Medicine, University of Warmia and Mazury, 10-045 Olsztyn, Poland; rtarg@op.pl (R.T.); piotr.cyganski@uwm.edu.pl (P.C.); paulina.nowek@uwm.edu.pl (P.N.); magdalena.starek@gmail.com (M.S.-S.); zajac.katarzyna@tlen.pl (K.Z.); 2 Department of Human Physiology and Pathophysiology, School of Medicine, University of Warmia and Masuria, 10-900 Olsztyn, Poland; judyta.juranek@uwm.edu.pl

**Keywords:** heart failure, common carotid intima-media thickness-to-lumen ratio, retinal arterioles wall-to-lumen ratio, arterial blood pressure

## Abstract

Current data indicate that heart failure (HF) is associated with inflammation and microvascular dysfunction and remodeling. These mechanisms could be involved in HF development and progression, especially in HF with preserved ejection fraction (HFpEF). We aimed to compare structural changes in retinal arterioles and carotid arteries between HF patients and patients without heart failure. This preliminary, retrospective, case-control study included 28 participants (14 patients with HFpEF and 14 age- and sex-matched healthy controls). Carotid intima-media thickness to lumen ratio (cIMTLR) was assessed using B-mode ultrasonography. Retinal arterioles wall- to-lumen ratio (rWLR) was assessed by adaptive optics camera rtx1. The HF patients had higher IMTLR (Δ_median_ [HFpEF–control group] 0.07, *p* = 0.01) and eWLR (Δ_median_ 0.03, *p* = 0.001) in comparison to patients without HF. In the whole study group, rWLR correlated significantly with IMTLR (r = 0.739, *p* = 0.001). Prevalence of arterial hypertension was similar in both groups, however, patients with HF had a significantly lower office, central and 24-h ambulatory blood pressure (systolic Δ_median_ −21 to −18 mmHg; diastolic Δ_median_ −23 to −10 mmHg). Our data suggests gradual and simultaneous progression of vascular remodeling in both retinal arterioles and carotid arteries in HFpEF patients. This process could be a marker of HF development. Significantly lower blood pressure values in HF group may indicate that vascular remodeling could be independent of BP control. Nevertheless, further and larger prospective studies allowing to reduce the impact of confounding and address temporality are warranted.

## 1. Introduction

The prevalence and hospitalizations related to heart failure with preserved ejection fraction (HFpEF) are rising [1]. To date, there are no effective therapies to reduce mortality in HFpEF. In part, it may depend on a lack of consensus on the basic pathophysiology, therapeutic targets and goals for therapy of this syndrome. The pathophysiology of HFpEF is related to cardiac structural and functional alterations. Arterial hypertension (AH) affects the risk of developing HFpEF, and antihypertensive treatment substantially lowers this risk [1,2]. Uncontrolled AH leads to vascular and cardiac remodeling. In patients with primary AH, the microcirculation is characterized by substantial structural and functional changes [3,4,5]. Small artery remodeling characterized by an increased wall-to-lumen ratio (WLR) is a distinctive feature of microvascular target organ damage in AH [4,6] and can be assessed noninvasively and in vivo by an adaptive optics camera in retinal arterioles [7]. Hypertensive patients with poor blood pressure (BP) control had a greater WLR of retinal arterioles than those with good blood pressure control [7,8]. A close correlation was observed between media to lumen ratio of subcutaneous small arteries and WLR of retinal arteries, indicating that a non-invasive, easily accessible evaluation provides similar information about microvascular morphology compared with accurate, but invasive, more labor-consuming and expensive micromyographic measurement of small subcutaneous arteries [9]. In greater arterial vessels such as the common carotid artery, elevated blood pressure plays a role in the increase of carotid intima-media thickness (cIMT) [10], considered to be a marker of target organ damage. Clinical trials of antihypertensive medications have shown cIMT regression among patients receiving effective therapy [11,12]. However, recently, it was demonstrated that good blood pressure control only slightly decreased but did not stop the progression of cIMT [13]. The heart is the most important target organ involved in the arterial AH. Left ventricular hypertrophy is associated with higher morbidity and mortality in hypertensive patients [14]. Even if the arterial AH is one of the most frequent causes of HFpEF, it is not clear if it is similar to those observed in hypertensive patients, such as the changes in micro- and macro-vascular structure which could be observed in HFpEF patients. Recent studies separately showed that HFpEF is associated with detrimental changes in retinal microcirculation and carotid arteries [15,16,17,18]. However, to our knowledge, no previous study has simultaneously evaluated retinal, carotid and left ventricle remodeling in patients with HFpEF.

The aim of this study was to compare the structure of small retinal arterioles, carotid arteries and the left ventricle between HFpEF and control patients using different evaluation methods and to establish whether a novel, simple, non-invasive method could be an alternative to routinely used invasive techniques.

## 2. Materials and Methods

### 2.1. Study Design

The present study was a single center, retrospective, case control. We have recruited 14 HFpEF patients (61–77 years old) and 14 age- and sex-matched controls without HF (65–76 years old). All heart failure group patients fulfilled the latest European Society of Cardiology criteria for heart failure diagnosis [19]. HFpEF group included HFpEF ambulatory patients stable for at least three consecutive months with NYHA class I-III, who have had a history of hospitalization due to heart failure decompensation in the Department of Cardiology and Internal Medicine. The control group included subjects with no previous history of cardiovascular diseases including HF recruited previously into a different study held in the Department. Exclusion criteria were as follows: refusal to give consent, inability to undergo adaptive optics camera assessment, heart failure exacerbation, acute coronary syndrome, acute infections in the last three months, uncontrolled thyroid and active neoplastic disorders.

The study was performed in accordance with the Declaration of Helsinki and the principles of ‘good clinical practice’ (GCP) guidelines. Approval of the local ethics committee was obtained (no. 595/15/Bioet) and informed consent was signed by all participants. 

### 2.2. Retinal Arteriolar Structural Assessment

Retinal arteriolar structural parameters were assessed using an adaptive optics retinal camera (rtx1; Imagine Eyes, Orsay, France). No pupil dilation was necessary. After a 5 min rest, patients were put on a chin rest. Briefly, the rtx1 camera measures and corrects wave front aberrations with a 750 nm super luminescent diode source and an AOC system operating in a closed loop. A 4° × 4° fundus area is illuminated at 840 nm by a temporally low coherent light-emitting diode flashed flood source and a stack of 20 fundus images is acquired in 2 s by a charged coupled device camera. Gaze was oriented using a dedicated target in order to capture the region of interest, which included a segment of the superotemporal artery of the right eye, devoid of bifurcations, at least 250 µm long and with an inner diameter of at least 50 µm. The site of interest was chosen to be free of the presence of neither focal arterial nicking nor arterio-venous crossings [4]. Based on collected measurements, a retinal arterioles wall-to-lumen ratio (rWLR) was calculated. All patients were examined in the same room with constant temperature of 23 °C, in darkness, in a sitting position. Retinal measurements were not completed for 4 participants from the HFpEF group because of cataracts (*n* = 20).

### 2.3. Carotid Artery Ultrasound

Common carotid artery ultrasound examinations were performed using the Sonoace R7 LN5-12 (Samsung, Korea) equipped with a linear probe (5–12 MHz). The procedure was carried out according to the Mannheim Intima-Media Thickness Consensus [20]. All subjects were examined in the same room with constant temperature of 23 °C, in dim light, in a sitting position after 5 min of rest. cIMT and lumen were measured at the end-diastole in the far walls of the left and right common carotid arteries 2 mm from the carotid sinus. cIMT measurements for each patient were expressed as a mean of cIMT. Then cIMT to lumen ratio (cIMTLR) for each subject was calculated. Just after carotid artery examination, arterial and central blood pressure (BP) were measured by AtCor Medical’s SphygmoCor^®^ (Sydney, Australia). 

### 2.4. 24 h Ambulatory Blood Pressure Monitoring

Patients from both study groups underwent 24 h ambulatory blood pressure monitoring (ABPM) within 1–2 weeks after enrolling in the study. All fully automatic monitors (Oscar 2™; SunTech Medical^®^/Morrisville, NC, USA) used the oscillometric technique and were programmed to take readings every 15 (daytime) or 30 min (night). All ABPM reports included in the study had 24 h monitoring with at least one valid measurement per hour. Blood pressure readings were automatically evaluated with a dedicated software (AccuWin Pro™ 4 from SunTech Medical^®^). A mean of 24 h recordings, a mean 24 h systolic BP (24 h SBP) and mean 24 h diastolic BP (24 h DBP) were collected for further evaluation. The differences between daytime SBP and night-time SBP (Dip-SBP) and between daytime DBP and night-time DBP (Dip-DBP) were calculated [21].

In accordance to ESH guidelines [22], we considered the patients to be hypertensive if their systolic BP (SBP) was ≥140 mm Hg and/or diastolic BP (DBP) ≥90 mm Hg in the repeated office measurements or otherwise if the AH was previously diagnosed and treated with antihypertensive drugs.

### 2.5. Transthoracic Echocardiography

Transthoracic echocardiography was performed by experienced sonographers using a digital commercial harmonic imaging ultrasound system (GE Vivid S5) with an S3 3-MHz phased-array transducer at a single center. Parasternal and apical 2-dimensional echocardiograms (2D) were acquired according to the American Society of Echocardiography/European Association of Echocardiography recommendations [23]. Finally, diastolic septum wall thickness (SWT), posterior left ventricle (LV) wall thickness (PWT) and the diameter of the left ventricle (LVDd) were measured by the 2D echo recordings. Left ventricle mass index (LVMI) was calculated using the following equations: LVMI = [0.8 × (1.04 × [LVDd + PWT + SWT]^3^ − [LVDd]^3^) + 0.6 g]/body surface area. Body surface area was calculated from the height and the weight according to Mosteller’s formula [24].

LV BSA = 0.007184 × W^0.425^ × H^0.725^

Relative wall thickness (LVRWT) was calculated by dividing the sum of SWT and PWT by the LVDd. LV ejection fraction (LVEF) was calculated using Simpson’s method [23]. Transmitral spectral Doppler was performed to obtain mitral inflow peak E-wave and peak A-wave velocities. Doppler Tissue Imaging Myocardial velocities were measured using a standard pulse-wave Doppler technique. The imaging angle was adjusted to ensure as near a parallel alignment of the beam as possible with the myocardial segment of interest. The sample volume was placed at the junction of the LV wall with the mitral annulus of the lateral myocardial segment from the apical four-chamber view. Peak velocities during early diastole (Em) were measured. Estimated LV filling pressure was derived from the ratio of transmitral E velocity to Em velocity.

### 2.6. Other Clinical Covariates

Blood tests were performed in a certificated professional clinical laboratory (ISO 9001:2008). Each patient’s medical history and treatment was recorded during the initial visit following the enrollment. 

### 2.7. Statistical Analysis

Due to the small study group and non-normal distribution of quantitative variables, they were presented as median and interquartile range (IQR). Categorical variables were presented as a number of subjects and percentage. For comparisons between case and control groups, Mann–Whitney U-test and Chi-Square test were used, wherever applicable. Correlation analyses were performed by calculating Spearman correlation coefficient. A value of two-sided *p* < 0.05 was considered as statistically significant. All analyses were performed using the Statistica 12.0 PL package (Starsoft Polska, Kraków, Poland).

## 3. Results

Demographic data and medical history were generally similar in both groups (Table 1). Despite that the prevalence of AH in both groups was similar, almost all hypertensive patients in the control group were newly recognized during the enrollment visit. 

All patients with HFpEF continued their prescribed therapy since discharge from the clinic following admission due to decompensated HF. The following medications were used: ACE-i in 86%, ARB in 7%, MRA in 57%, β-blockers in 93%, loop diuretics in 86%, thiazide diuretic in 36% and statin in 57% of patients for more than 3 months prior to the enrollment in our study. In the control group without heart failure, one patient received ACE-i and another one received statin. 

The median values of office blood pressure measured at the study site in the HFpEF group were in the normal range whereas in the control group, the values of systolic and diastolic BP were significantly higher (SBP Δ_median_ (difference in median between case and control groups) −18 mmHg and DBP Δ_median_ −22 mmHg, *p* = 0.01 and *p* = 0.001, respectively). Additionally, central blood pressure measured during the ambulatory visit was significantly lower in HFpEF patients compared to the control group (central SBP Δ_median_ −21 mmHg and 24-hr central DBP Δ_median_ −23 mmHg, *p* = 0.001 both). Similarly, 24 h BP measurements were within normal values and significantly lower in the HFpEF group in comparison to controls without heart failure (24 h SBP Δ_median_ −20 mmHg and 24 h DBP Δ_median_ −22 mmHg, *p* = 0.01 and *p* = 0.02, respectively). Moreover, the HFpEF patients were characterized by significantly lower night BP decrease compared to controls (Dip-SBP Δ_median_ −7.0 mmHg and Dip-DBP Δ_median_ −10.8 mmHg, *p* = 0.02 and *p* = 0.04, respectively, Table 2). 

HFpEF patients had lower concentrations of Hb (Δ_median_ −1.7 pg/mL, *p* = 0.02), serum sodium (Δ_median_ −3 mmol/L, *p* < 0.02), cholesterol (Δ_median_ −16 mg/mL, *p* < 0.01) and higher concentrations of serum creatinine (Δ_median_ 0.1 mg/dL, *p* < 0.03), hsTnT (Δ_median_ 0.009 pg/mL, *p* < 0.001) and NT-proBNP (Δ_median_ 523 pg/mL, *p* < 0.002) compared to controls. The most relevant clinical and laboratory variables are presented in Supplementary Table S1.

Retinal examination, carotid ultrasound and echocardiographic parameters are presented in Table 3. Ultrasound imaging examinations revealed that HFpEF patients had increased markers of micro- and macrovascular remodeling compared to controls. In retinal arterioles, rWLR was significantly higher in heart failure group (Δ_median_ 0.07, *p* = 0.01). Similarly, in common carotid arteries of HFpEF patients, we found a significantly higher diameter of cIMT (Δ_median_ 0.02 cm, *p* = 0.004) and a significantly higher cIMTLR (Δ_median_ 0.03, *p* = 0.001). HFpEF patients had a significantly higher left atrium diameter (Δ_median_ 0.5 cm, *p* = 0.03) and lower LVEF (Δ_median_ −9%, *p* = 0.01) compared to controls. Left ventricular relative wall thickness and left ventricular mass index were similar in both studied groups (*p* = 0.3 and *p* = 0.9, respectively).

**Figure 1:**
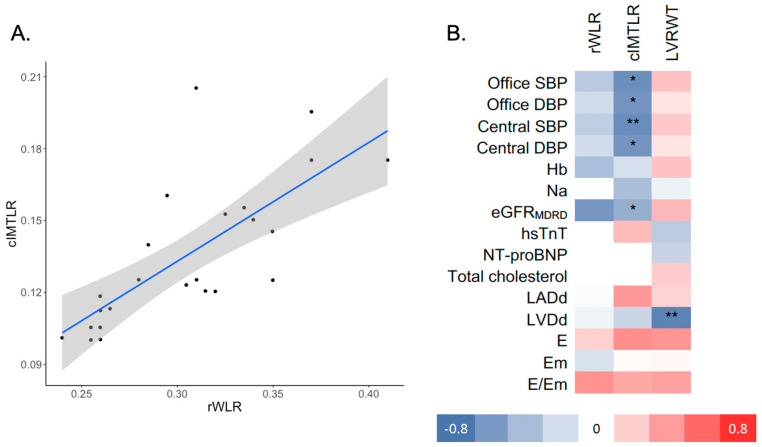
(**A**) Scatterplot presenting correlation between rWLR and cIMTLR and (**B**) heatmap of Spearman correlation coefficients between rWLR, cIMTLR, LVRWT and selected clinical covariates. * *p* < 0.05, ** *p* < 0.01; rWLR—retinal wall-to-lumen ratio; cIMTLR- carotid intima to media ratio; LVRWT—left ventricle relative wall thickness; Hb—hemoglobin; eGFR_MDRD_—estimated glomerular filtration rate; hsTnT—high-sensitivity troponin T; NT-proBNP—N-terminal prohormone of brain natriuretic peptide; LADd—left atrium diameter (diastolic); LVDd—left ventricle diameter (diastolic); LVRWT—left ventricle relative wall thickness; LVMI—left ventricle mass index; LVEF—LV ejection fraction; E—early diastolic mitral inflow velocity; Em—early diastolic mitral annular velocity.

rWLR (Figure 1A) was strongly correlated with cIMTLR (ρ = 0.74, *p* = 0.001). cIMTLR was inversely correlated with office SBP/DBP, central SBP/DBP and eGFR (ρ from −0.67 to −0.48, all *p* < 0.05, Table 3). Relative left ventricular wall thickness was inversely correlated with left ventricle diameter (ρ = −0.72, *p* = 0.001). 

## 4. Discussion

To our best knowledge, this is the first study of the retinal WLR estimated by adaptive optics camera rtx1 in patients with HFpEF. Our study’s most important finding is that the HFpEF patients had higher values of the retinal arterioles and common carotid arteries remodeling parameters when compared with age- and sex-matched controls. 

### 4.1. Large Artery Remodeling and Heart Failure

AH was the most frequent disorder present in both studied groups. It was established that AH is a major risk factor for HFpEF and that good blood pressure control substantially lowers this risk [25]. Increased blood pressure plays a major role in the intima-media thickening and left ventricular hypertrophy [10,14]. Although the frequency of AH prevalence was similar in both groups, carotid intima-media thickness was higher in subjects with HFpEF. Moreover, compared to controls, they had lower systolic and diastolic blood pressure values measured during the ambulatory visit and during 24 h ABPM. Lower values of BP in the HFpEF group in our study were due to the strict antihypertensive therapy, as opposed to the control group, where AH was diagnosed mainly during the study’s enrollment visit. We suppose that HFpEF patients had a much longer history of AH than controls without heart failure. Therefore, in this case, the use of antihypertensive medication could likely explain the inverse correlations of BP with cIMTLR. Antihypertensive treatment has been reported to reduce cIMT in hypertensive patients [26]. However, recent evidence shows that well-controlled blood pressure may only slightly decrease but not stop the progression in cIMT, which is likely to reflect hypertrophy of the arterial media layer and suggests that the increase in cIMT is not reversible in all cases [13]. Retinal and carotid arteries’ wall remodeling process, partially independent from AH control, may indicate a parallel mechanism contributing to vascular remodeling and potentially heart failure development. It was documented that increasing cIMT was associated with incident HF beyond risks explained by major CVD risk factors and CHD, which suggests that cIMT may be associated with HF beyond hypertension and myocardial ischemia [27]. Increased cIMT and the presence of atheromatous plaques are forms of carotid artery disease that are both regarded as surrogate markers of atherosclerosis. Although these two phenotypes are related, the pathophysiology underlying intima-media thickening and plaque formation are different. Hypertrophy of the media layer of the arterial wall can occur either as a response to hypertension or as a manifestation of aging. In contrast, plaque formation represents the maturation of the atherosclerotic process [28]. The mean age in patients from our study was over 65 years in both groups. Elderly patients with HF differ from younger patients with HF in terms of several characteristics, including the relatively large proportion of HFpEF in the elderly population [2,29]. The Cardiovascular Health Study, which included subjects 65 years of age or older, revealed that atherosclerosis, as measured by cIMT, predicts systolic and diastolic HF as more prevalent in older patients [30].

### 4.2. Small Artery Remodeling and Heart Failure

In HFpEF patients, we found higher values of WLR in the small retinal arterioles compared to the control group. Moreover, these changes significantly positively correlated with IMTL ratio in the whole study group. AH, the most frequent comorbidity in our study, is an important cause of remodeling retinal arterioles [30]. Previous studies reported that hypertensive patients with poor BP control had a greater WLR of retinal arterioles than those with good BP control [7,8]. Moreover, in the cohort of never-treated patients with essential hypertension and normotensive controls, both SBP and DBP were significantly linked to WLR of retinal arterioles and independent of traditional cardiovascular risk factors [31]. Our study revealed that HFpEF patients, who had lower values of BP during ambulatory visit and 24 h ABPM, had higher WLR in comparison to hypertensive controls without heart failure. We could not rule out that other factors, such as the duration of AH or other processes associated with the HFpEF development, could be responsible for the observed changes in retinal vessels in our study. Previously, it was reported that retinopathy is an independent predictor of HF, even in people without preexisting coronary heart disease, diabetes, or hypertension [32]. This suggests that microvascular disease may play an important role in the development of heart failure in the general population [32]. These data were confirmed by 18-year-long observational studies of ARIC patients without cardiovascular disease, where structural changes of retinal arterioles such as narrower central retinal arteriolar equivalent was significantly and linearly associated with future incident HF and was a simple, non-invasive test that predicted HF and adverse cardiac structure/function for up to 18 years in the future [33]. However, there are studies indicating that other microvascular changes could be more specific to HFpEF. It was found that microvascular abnormalities demonstrated by videodermatoscopic examination of nailfold capillaries are considerably more common in HFpEF patients compared to HFrEF and control groups [34].

### 4.3. Limitations

There are several limitations, which have to be taken into consideration while interpreting our results. First of all, this study has a preliminary character. Therefore, some of the described comparisons could potentially be underpowered. Secondly, subjects from cases and control groups were recruited from groups of patients with a number of differences in their clinical characteristics, as well as previous medical history. Due to single center recruitment and a small pool of potential participants resulting in small sample size, we could not adopt a potential study design solution that could improve the comparativeness of studied groups and lower the likelihood of selection bias. In particular, we could use propensity score in our analysis. Due to a low number of observations per variable, a meaningful propensity score could not be constructed as it would not include all important predictors of HFpEF. Moreover, we cannot exclude the influence of residual confounding. Despite no significant differences in patients’ characteristics between groups, we were not able to collect reliable data on the length of HFpEF or AH. It can be just assumed that the HFpEF group had a longer history of BP, which contributed to the development of HF. The following evidence for micro- and macrovascular remodeling could also be attributed to atherosclerosis. Despite that only four subjects had coronary heart disease (as approximated by history of MI, CABG and PCI), being low, as patients did not undergo arteriography, we could not exclude subclinical atherosclerosis. Median cIMT values in this study corresponded to top decile for this age group. Lastly, due to the cross-sectional character of the analysis, we could not assess the temporality of associations, nor adjust for time-variant factors such as treatment. This issue led to paradoxical findings such as inverse correlations between BP and cIMTLR, which are explained by medical history rather than by reverse causation. 

## 5. Conclusions

We found that heart failure patients with preserved ejection fraction had significantly higher cIMTLR and retinal arterioles WLR in comparison to the control group without heart failure. There was a significant positive correlation between cIMTLR and retinal arterioles WLR ratios in the whole study group. This might suggest parallel gradual progression of vascular remodeling in both retinal arterioles and carotid arteries, which could impact HFpEF development. The pathophysiological mechanism responsible for this correlation needs further evaluation, however, significantly lower blood pressure values in the HFpEF group may indicate that vascular remodeling is independent of blood pressure control. Nevertheless, further and larger prospective studies allowing to reduce impact of confounding and address temporality are warranted.

## Figures and Tables

**Table 1:** Baseline characteristics of study participants.

Variable	HFpEF Group *n* = 14	Control Group *n* = 14	*p*
Age (years)	73 (61–77)	69 (66–75)	0.7
Men (%)	4 (29%)	4 (29%)	1.0
Body mass index (kg/m^2^)	32.8 (26.0–36.9)	27.8 (22.2–28.7)	0.05
Arterial hypertension	13 (93%)	12 (86%)	0.6
Previous MI, PCI or CABG	4 (29%)	0 (0%)	0.1
Atrial fibrillation	4 (79%)	0 (0%)	0.1
Diabetes mellitus	5 (36%)	1 (7%)	0.1
COPD	3 (21%)	0 (0%)	0.2
Active smoker	8 (57%)	7 (50%)	0.7

Categorical variables are presented as number (% of group), while continuous as median [interquartile range]. *p* stands for chi-square (categorical) or Mann–Whitney U-test (continuous). HFpEF—heart failure with preserved ejection fraction; MI—myocardial infarction; PCI—percutaneous coronary artery intervention; CABG—coronary artery bypass graft; COPD—chronic obstructive pulmonary disease.

**Table 2:** Differences in blood pressure measured using different techniques between HFpEF and control groups.

Variable	HFpEF Group *n* = 14	Control Group *n* = 14	Δ_median_	*p*
Heart rate (1/min)	61 (56–65)	69 (58–73)	−8	0.1
Office SBP (mmHg)	130 (122–131)	148 (145–161)	−18	0.01
Office DBP (mmHg)	72 (66–79)	94 (89–100)	−22	0.001
Central SBP (mmHg)	114 (111–123)	135 (132–144)	−21	0.001
Central DBP (mmHg)	73 (68−80)	96 (89–101)	−23	0.001
24 h ambulatory blood pressure monitoring				
24 h SBP (mmHg)	109 (109–118)	129 (124–133)	−20	0.01
24 h DBP (mmHg)	64 (60–66)	74 (67–78)	−10	0.02
Dip-SBP (mmHg)	2.5 (−2.9–5.3)	9.5 (5.0–14.8)	−7.0	0.02
Dip-DBP (mmHg)	5.9 (0.9–2.3)	16.7 (7.9–23.8)	−10.8	0.04

Values are expressed as median and interquartile range. Δ stands for difference in median between HFpEF and control groups. *p* stands for Mann–Whitney U-test. HFpEF—heart failure with preserved ejection fraction; SBP—systolic blood pressure; DBP—diastolic blood pressure; Dip-(S/D)BP—differences between daytime and night-time SBP/DBP.

**Table 3:** Differences in retinal, carotid and echocardiographic markers between HFpEF and control groups.

Variable	HFpEF Group *n* = 14	Control Group *n* = 14	Δ_median_	*p*
Retinal arteriolar structural assessment				
rWLR	0.34 (0.31–0.37)	0.27 (0.26–0.31)	0.07	0.01
Carotid artery ultrasound				
cIMT (cm)	0.10 (0.09–0.11)	0.08 (0.07–0.08)	0.02	0.004
cL (cm)	0.61 (0.59–0.73)	0.67 (0.57–0.73)	−0.06	0.9
cIMTLR	0.15 (0.13–0.18)	0.12 (0.11–0.13)	0.03	0.001
Transthoracic echocardiography				
LADd (cm)	4.4 (4.1–4.8)	3.9 (3.7–4.1)	0.5	0.03
LVDd (cm)	5.1 (4.8–5.5)	4.8 (4.5–5.3)	0.3	0.3
LVRWT (cm)	0.41 (0.38–0.44)	0.47 (0.39–0.48)	−0.06	0.3
LVMI (g/m^2^)	101.0 (89.3–110.0)	99.3 (92.5–119.0)	1.7	0.9
LVEF (%)	58 (50–62)	67 (63–71)	−9	0.01
E	64 (49–93)	65 (56–68)	−1	0.9
Em	7 (6–9)	8 (7–11)	−1	0.7
E/Em (lateral)	8.9 (7.1–13.3)	7.9 (6.3–8.6)	1.0	0.3

Values are expressed as median and interquartile range. Δ stands for difference in median between HFpEF and control groups. *p* stands for Mann–Whitney U-test. HFpEF—heart failure with preserved ejection fraction; rWLR—retinal wall-to-lumen ratio; cIMT—carotid intima-media thickness; cL—carotid lumen diameter; cIMTLR—carotid intima to media ratio; LADd—left atrium diameter (diastolic); LVDd—left ventricle diameter (diastolic); LVRWT—left ventricle relative wall thickness; LVMI—left ventricle mass index; LVEF—LV ejection fraction; E—early diastolic mitral inflow velocity; Em—early diastolic mitral annular velocity.

## Data Availability

The data presented in this study are available on request from the corresponding author. The data are not publicly available due to ongoing unpublished research.

## Supplementary Materials

The following supporting information can be downloaded at: https://www.mdpi.com/article/10.3390/jcm11133721/s1, Table S1: Differences in retinal, carotid, and echocardiographic markers between HFpEF and control groups.
