# Supplementary material for: Remodeling of Retinal Arterioles and Carotid Arteries in Heart Failure Development—A Preliminary Study
Source: J Clin Med. 2022 Jun 27;11(13):3721. doi: 10.3390/jcm11133721 (PMC9267807; doi:10.3390/jcm11133721)
Supplement: Supplementary file 1 [file jcm-11-03721-s001.zip › jcm-1717654-supplementary.pdf]

Supplementary Table S1. Differences in retinal, carotid, and echocardiographic markers between HFpEF and control groups.

**Variable**	**HFpEF group** ***n*=14**	**Control group** ***n*=14**	Δ_{median}	***P***
Hemoglobin (g/dl)	12.4 [11.9-14.0]	14.1 [13.6-16.0]	-1.7	**0.02**
Na (mmol/L)	140 [138-142]	143 [142-144]	-3	**0.02**
Creatinine (mg/dl)	0.9 [0.9-1.2]	0.8 [0.7-0.9]	0.1	**0.03**
eGFR (ml/min)	62.1 [44.9-79.5]	83.3 [79.5-98.1]	-21.2	**0.01**
hsTNT(ng/ml)	0.014 [0.011-0.019]	0.005 [0.003-0.007]	0.009	**<0.001**
NT-proBNP (pg/ml)	616 [185-2854]	93 [55-159]	523	**0.002**
Total cholesterol (mg/dl)	170 [160-187]	186 [180-228]	-16	**0.009**
LDL-C (mg/dl)	88 [69-92]	101 [74-154]	-13	0.3
Triglicerides (mg/dl)	95 [70-132]	107 [901-146]	-12	0.4

Values are expressed as median and interquartile range. Δ stands for difference in median between HFpEF and control groups. *P* stands for Mann–Whitney U-test. HFpEF – heart failure with preserved ejection fraction; $eGFR_{MDRD}$ – estimated glomerular filtration rate; hsTnT – high-sensitivity troponin T; NT-proBNP – N-terminal prohormone of brain natriuretic peptide; LDL-C – low-density lipoprotein cholesterol.
